# TNF-α-induced Inflammation Stimulates Apolipoprotein-A4 via Activation of TNFR2 and NF-κB Signaling in Kidney Tubular Cells

**DOI:** 10.1038/s41598-017-08785-2

**Published:** 2017-08-18

**Authors:** Hyung Ho Lee, Young In Cho, Sook Young Kim, Young Eun Yoon, Kyung Sup Kim, Sung Joon Hong, Woong Kyu Han

**Affiliations:** 1 Department of Urology, Urological Science Institute, Yonsei University College of Medicine, Seoul, 120-752 Korea; 2 Brain Korea 21 PLUS Project for Medical Science, Yonsei University College of Medicine, Seoul, 120-752 Korea; 3 Department of Biochemistry and Molecular Biology, Institute of Genetic Science, Integrated Genomic Research Center for Metabolic Regulation, Yonsei University College of Medicine, Seoul, 120-752 Korea; 4 Department of Urology, National Health Insurance Service Ilsan Hospital, Gyeonggi-do, 10444 Korea; 5 Department of Urology, Hanyang Univesity College of Medicine, Seoul, 04763 Korea

## Abstract

Apo-A4 expression was increased in tissues from chronic kidney disease (CKD) patients compared to that in normal kidney tissue. We determined the association of apo-A4 and its regulatory signals following acute kidney injury and elucidated the effects of apo-A4 on cell signaling pathways related to kidney injury *in vitro* and *in vivo*. Tumor necrosis factor (TNF)-α, which causes inflammatory cell injury, induced significantly increased expression of apo-A4 protein levels, and these levels were related to pro-inflammatory acute kidney injury in human kidney cells. Apo-A4 expression was also increased in experimented rat kidney tissues after ischemic reperfusion injury. The expression of tumor necrosis factor receptor (TNFR) 2 was increased in both kidney cell lines and experimented rat kidney tissues following acute kidney injury. The expression of apo-A4 and TNFR2 was increased upon treatment with TNF-α. Immunohistochemistry revealed positive apo-A4 and TNFR2 staining in ischemic reperfusion injury rat kidneys compared with levels in the sham operation kidneys. After neutralization of TNF-α, NF-κB expression was only observed in the cytoplasm by immunofluorescence. Therefore, the apo-A4 expression is increased by stimulation of injured kidney cells with TNF-α and that these effects occur via a TNFR2-NFκB complex.

## Introduction

In human kidney, apo-A4 is localized to the proximal and distal tubular cells. Apo-A4 is filtered by the glomeruli in its lipoprotein-unbound state and then taken up by proximal tubular cells for degradation^1^. Apo-A4 is associated with several kidney diseases (nephrotic syndrome vs. non-nephrotic syndrome), and expression in kidney tubular cells suggests that apo-A4 is metabolized in the kidney^2, 3^. Creatinine clearance correlates negatively with serum apo-A4 expression in kidney diseases. Moreover, patients with a tubular type of proteinuria have significantly higher amounts of apo-A4 in their urine than those with a pure glomerular type of proteinuria and controls^4^. Apo-A4 levels increase significantly with decreasing glomerular filtration rates and are elevated in early stages of chronic kidney disease (CKD). Higher concentrations of plasma apo-A4 serve as a good predictor for the progression of kidney disease^5, 6^. Therefore, apo-A4 levels represent a novel predictor for kidney disease progression^3^. Elevated apo-A4 levels in response to impaired kidney function may enhance the removal of cholesterol from mesangial cells, but this has yet to be confirmed^7^. Significantly higher apo-A4 levels are observed in the blood plasma and urine of CKD patients, and apo-A4 accumulation increases significantly during the initial phase of the disease^2, 8^. Continuous apo-A4 accumulation following kidney injury leads to amyloidosis, which is characterized by amyloid deposits in body tissues and organs^4^. Therefore, apo-A4 accumulation may have an adverse impact on in kidney cell recovery. These findings suggest that apo-A4 levels increase as kidney injury progress and that apo-A4 influences the recovery of injured kidney cells. To date, the molecular signals involved in the association between apo-A4 and kidney injury have not been investigated. Therefore, the aim of this study was to demonstrate the association of apo-A4 and regulatory signals of apo-A4 following acute kidney injury (AKI) and elucidate the effects on cell signaling pathways related to kidney injury *in vitro* and *in vivo*.

## Results

### Localization and expression of apo-A4 in kidney cell lines and human kidney tissues

**Figure 1:**
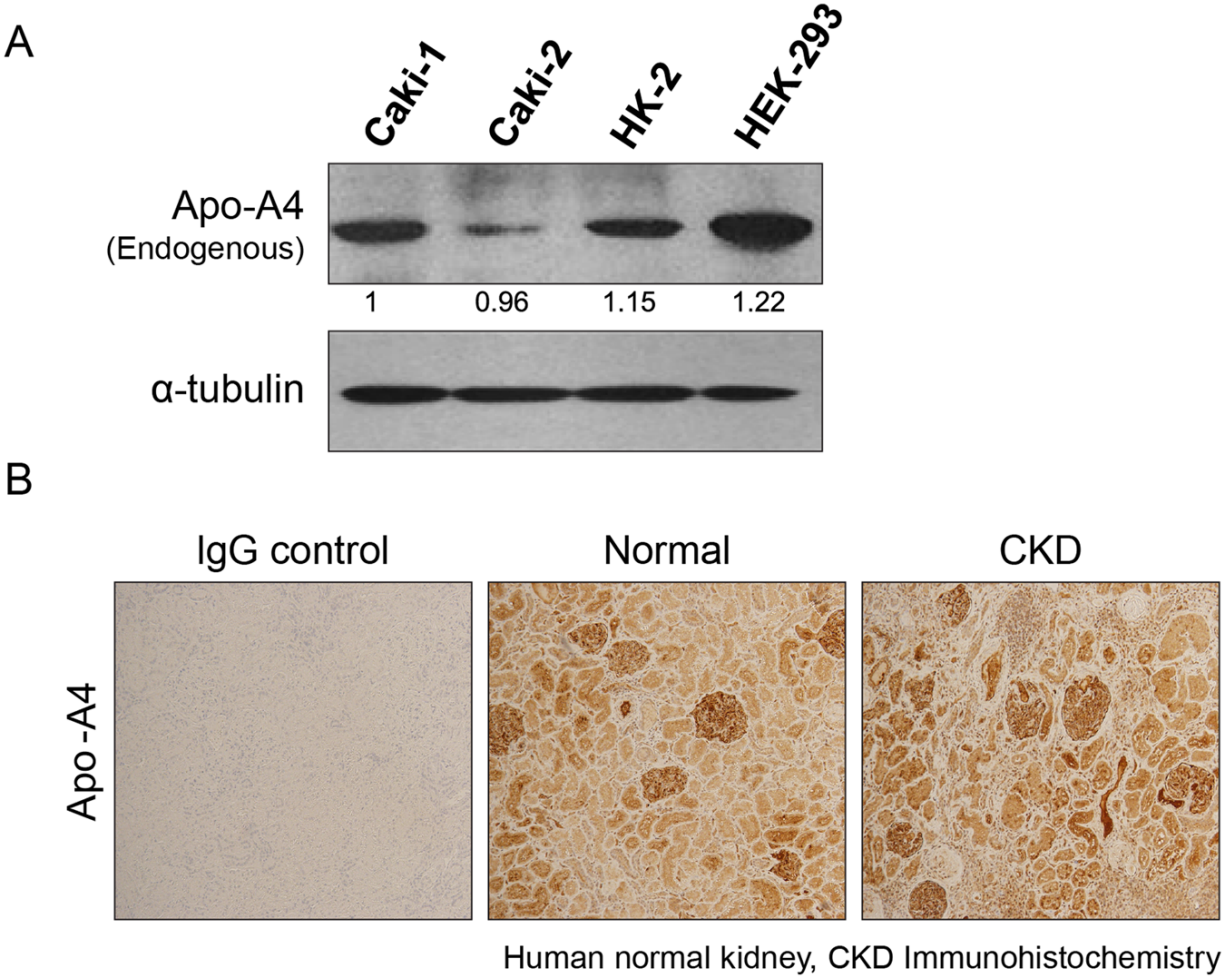
Kidney cell lines and human kidney tissues expressed apo-A4. (**A**) Endogenous apo-A4 protein expression was measured in Caki-1, Caki-2, HK-2, and HEK-293 cells by western blotting. The loading control was α-tubulin. (**B**) IHC of apo-A4 in Formalin-fixed, paraffin-embedded human normal and CKD kidney tissue.

To investigate the expression of apo-A4 in the kidney, we used two kidney cancer cell lines (caki-1 and caki-2) and two normal kidney cell lines (HK-2 and HEK-293). We measured apo-A4 expression levels in these cells using western blotting (Fig. 1A). We observed apo-A4 protein expression in all four kidney cell lines. Also, we examined apo-A4 expression in normal kidney and CKD tissues by immunohistochemistry (IHC) using a mouse anti-apo-A4 antibody. IHC revealed apo-A4 expression in normal and CKD tissues (Fig. 1B). We detected apo-A4 expression in the tubules as well as in the glomeruli, and interestingly, the intensity of apo-A4 expression in CKD tissue was stronger than in healthy kidney tissue.

### Apo-A4 expression in kidney cell lines following renal injury model

**Figure 2:**
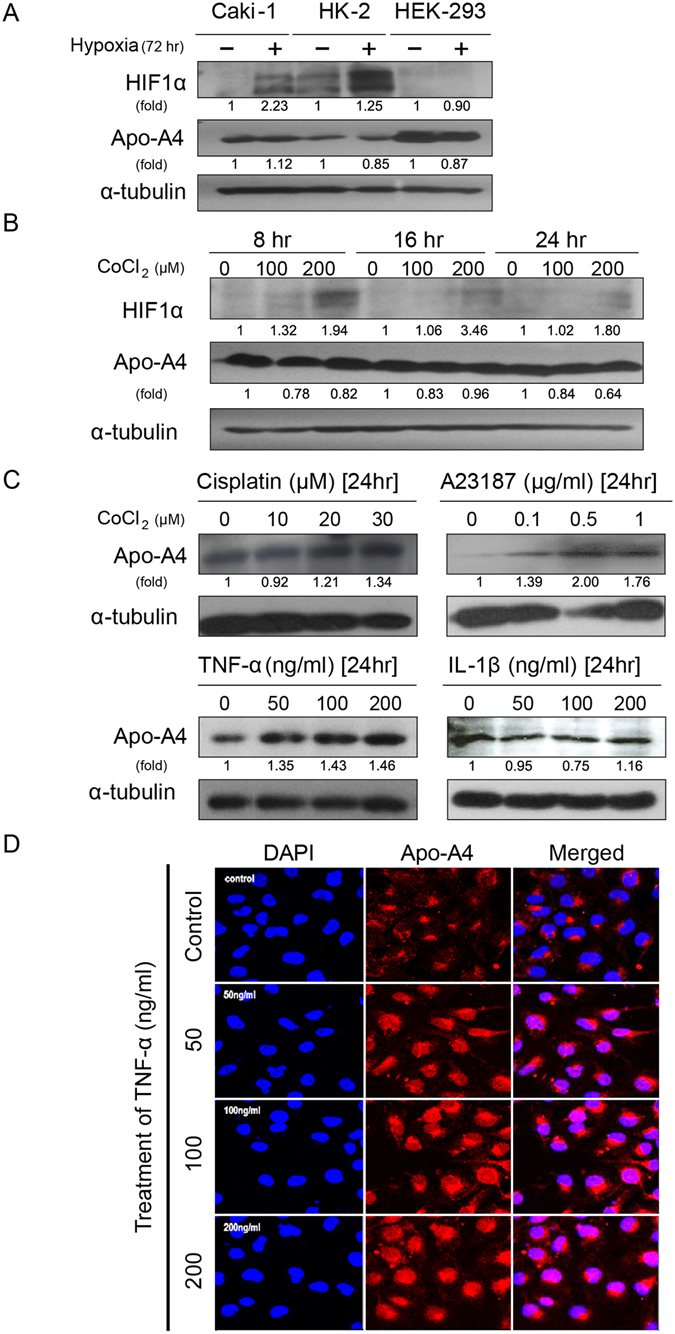
Effect of renal injury model expressed apo-A4 in kidney cell lines. (**A**) Hypoxic cell injury; HIF-1α and apo-A4 expression in Caki-1, HK-2, and HEK-293 cells exposed to hypoxia for 72 h. (**B**) Chemical cell injury; HIF-1α and apo-A4 expression in HK-2 cells treated with different concentrations of CoCl_2_ (0, 100, and 200 µM) for 8, 16, and 24 h. (**C**) Cytotoxic cell injury and cytokaine cell injury; Apo-A4 expression in HK-2 cells treated with cisplatin (0, 10, 20, and 30 µM), A23187 (0, 0.1, 0.5, and 1 μg/ml), TNF-α (0, 50, 100, and 200 ng/ml), and IL-1β (0, 50, 100, and 200 ng/ml) for 24 h (see Supplementary Figs S1, 2 and 3). (**D**) Immunofluorescence assay showing localization of apo-A4 (red) in HK-2 cells after 24 h of treatment with TNF-α.

To determine which type of renal injury model affects apo-A4 expression in kidney cells, we exposed human kidney cells to conditions known to induce an AKI. We incubated Caki-1, HK-2, and HEK293 cells in a hypoxic chamber for 72 h (Fig. 2A); yet, we detected no significant expression of apo-A4 in any of these cell-lines as a result. We induced chemical hypoxia using CoCl_2_ (0, 100, and 200 µM) for 8, 16, and 24 h in HK-2 cells, but these conditions also had no effect on apo-A4 expression in these cell types (Fig. 2B). Also, we treated HK-2 cells with the nephrotoxic cancer drug cisplatin; the calcium ionophore A23187, which induces calcium influx that causes apoptosis; and the pro-inflammatory cytokines TNF-α and IL-1β, which are known to cause inflammatory injury^9, 10^. Following treatment, we measured apo-A4 protein expression levels by western blotting (Fig. 2C). While cisplatin and IL-1β failed to modify the expression of apo-A4 in these cells, TNF-α induced significantly increased expression of apo-A4. Although we observed an increase in apo-A4 following treatment with A23187, this change was not significant. Immunofluorescence assays revealed an increase in apo-A4 expression 24 h after TNF-α treatment compared with levels in the untreated control group (Fig. 2D). Furthermore, the expression of apo-A4 in the nucleus of HK-2 cells was increased in accord with TNF-α treatment. Thus, of the renal injury agents tested, only TNF-α significantly affects the expression of apo-A4.

### Inflammatory AKI induced by TNF-α increased apo-A4 Expression

**Figure 3:**
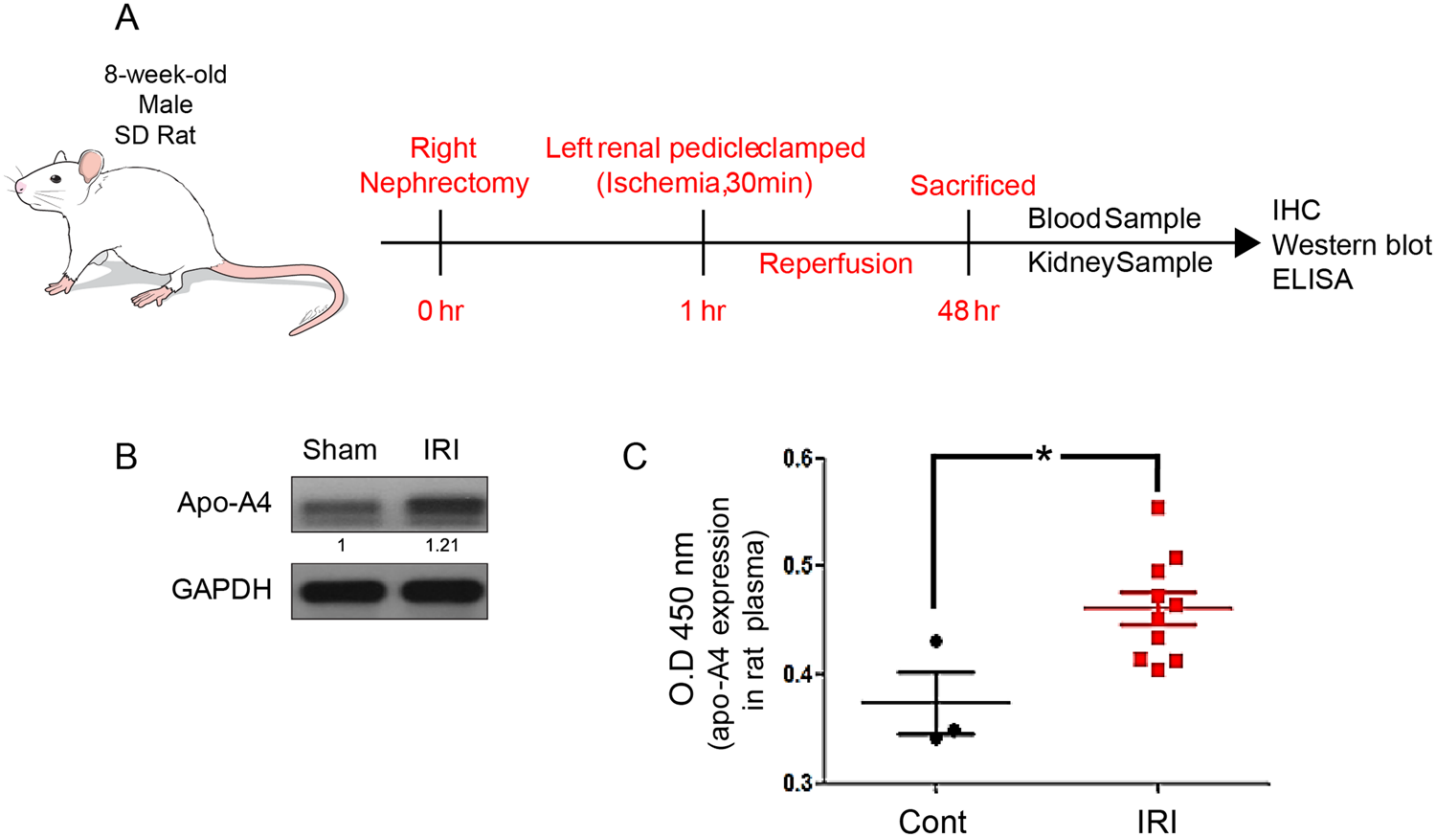
Inflammatory AKI induced by TNF-α increased apo-A4 expression *in vivo*. (**A**) Graphical presentation of *in vivo* experiment method with male SD rat. (**B**) Expression of apo-A4 protein detected by western blotting of kidney tissue from an ischemic reperfusion injury rat model. (**C**) Apo-A4 expression in rat plasma measured by ELISA. Results represent the mean (±SEM) of ten independent experiments (n = 10). The statistical signification was marked as * for p < 0.05 compared with the control.

To examine whether AKI induced by TNF-α increased apo-A4 expression *in vivo*, we induced ischemic reperfusion injury in rat kidneys (Fig. 3A)^7^. We removed kidney tissue 48 h after reperfusion and isolated protein lysates to measure apo-A4 expression levels by western blotting. Apo-A4 protein expression was elevated in ischemic reperfusion injury tissue compared with levels in control tissue (Fig. 3B). Also, we isolated serum samples from these rats and confirmed the increased apo-A4 levels by enzyme-linked immunosorbent assay (ELISA). Data presents 10 samples, and were shown as mean ± SEM and analyzed by students t-test (*P ≤ 0.05; Fig. 3C). Together, these findings indicate that apo-A4 expression is increased by inflammatory AKI *in vitro* and *in vivo*.

### Inflammatory AKI induced by TNF-α increases apo-A4 via TNF Receptor 2 (TNFR2)

**Figure 4:**
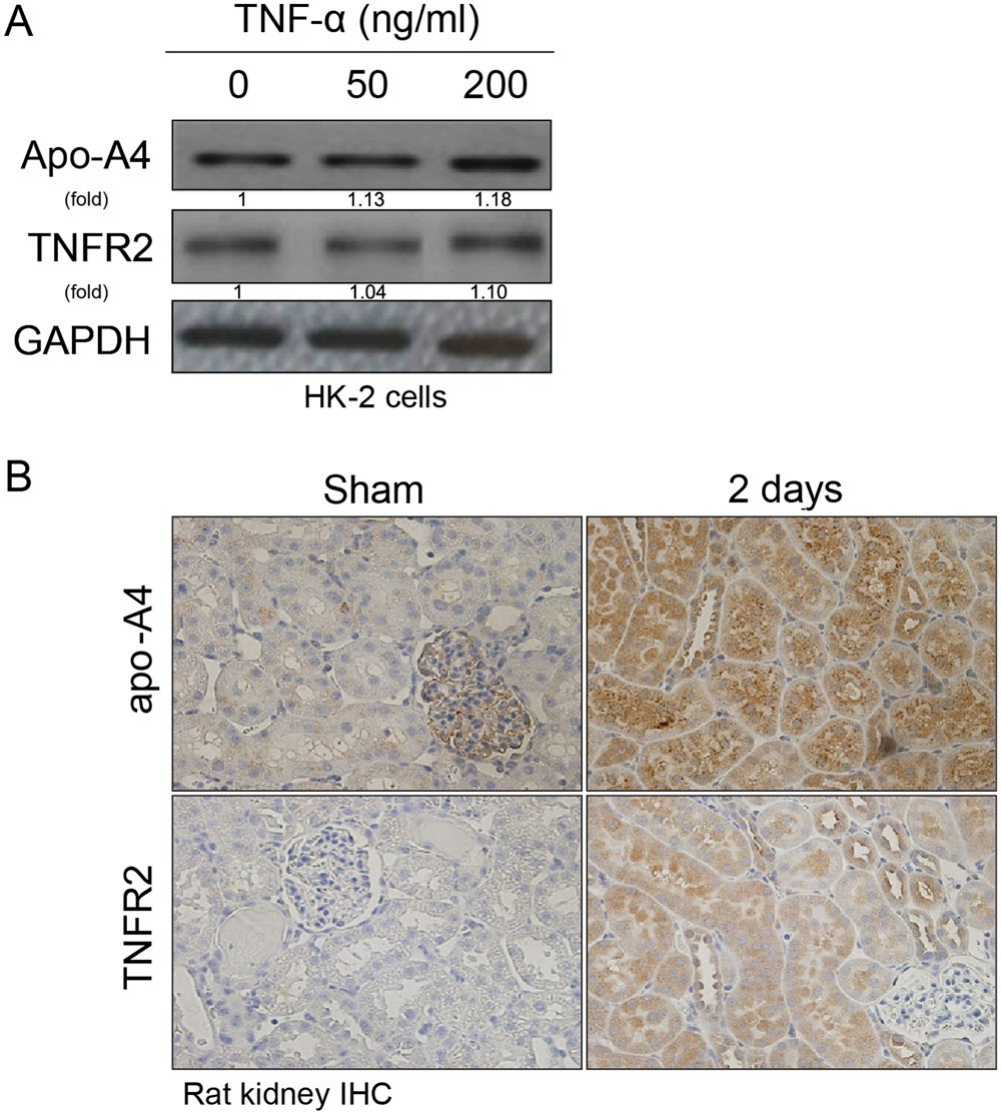
Inflammatory AKI induced by TNF-α increased apo-A4 expression via TNFR2. (**A**) HK-2 cells were treated with different concentrations of TNF-α (0, 50, and 200 ng/ml) for 24 h. Apo-A4 protein expression was detected by western blotting. (**B**) Apo-A4 and TNFR2 immunohistochemistry on formalin-fixed, paraffin-embedded tissue from ischemic reperfusion injury rat kidneys.

Expression of TNFR2 increases in tubular epithelial cells during ischemic reperfusion injury^11^. Therefore, we measured the expression of apo-A4 and TNFR2 upon induction of AKI. The expression of apo-A4 and TNFR2 were increased upon treatment of TNF-α as assessed by western blotting (Fig. 4A). Also, we detected the expression of apo-A4 and TNFR2 by IHC in rat kidney tissue. Furthermore, IHC revealed positive apo-A4 and TNFR2 staining in ischemic reperfusion injury rat kidneys compared with the sham operation kidneys (Fig. 4B). These experiments revealed a positive correlation between the expression of apo-A4 and TNFR2 following treatment with the pro-inflammatory cytokine TNF-α.

### Increased apo-A4 expression caused by inflammatory AKI is associated with the NF-κB Complex

**Figure 5:**
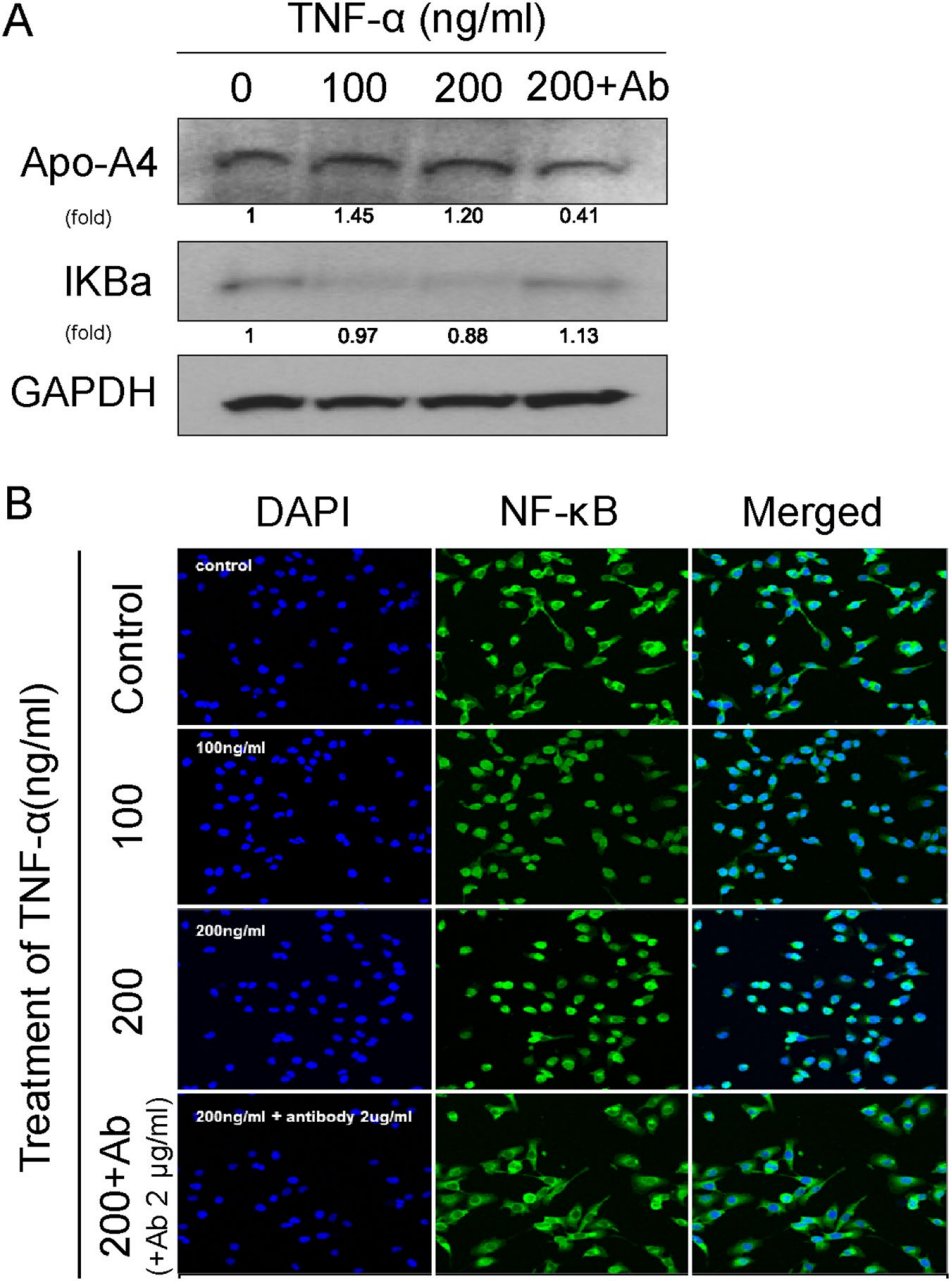
Inflammatory AKI increases apo-A4 expression through the NF-κB complex. (**A**) Western blotting revealed an inverse relationship between apo-A4 and IκB protein expression in HK-2 cells following TNF-α treatment. The loading control was GAPDH and the normalized ratio of each extract pair (A set to 1) determined using Image Jv1.42q software (http://rsb.info.nih.gov/ij). **(B**) Immunofluorescence assay for apo-A4 and NF-κB expression in HK-2 cells after 24-h TNF-α treatment. TNF-α antibody was used to neutralize the TNF-α effect. NF-κB (green) immune-localization is shown. And NF-κB regulates apo-A4 (see Supplementary Fig. S4).

The NF-κB complex plays a major role in inflammation^12^. We treated HK-2 cells with TNF-α, TNF-α antibody and then measured IκB and apo-A4 expression by western blotting. We observed an inverse correlation between apo-A4 and IκB expression levels (Fig. 5A). Western blotting revealed expression in apo-A4 increased by TNF-α concentration. When 100, 200 ng/ml of TNF-α were administered, apo-A4 increased by 1.45 and 1.2. Moreover, apo-A4 levels decreased significantly to 0.41 for TNF-α antibody treated group. The expression of IκB was similar to control when treated TNF-α antibody (Fig. 5A). Also, we neutralized TNF-α using a specific TNF-α antibody and then examined NF-κB localization using an immunofluorescence assay. NF-κB expression was only observed in the cytoplasm after neutralization of TNF-α in comparison with localization of this cytokine in untreated HK-2 cells (Fig. 5B). This finding suggests that NF-κB translocation is promoted by TNF-α, which elevates apo-A4 expression levels.

## Discussion

AKI occurs in the initial stages of kidney disease and is followed by CKD^13^. Previous work has demonstrated that apo-A4 expression in kidney was significantly elevated during AKI and that serum apo-A4 increases during the earliest phases of renal insufficiency^3–5^. We had previously shown that apo-A4 expression levels are strongly reduced in pig kidneys when ischemic preconditioning was performed prior to renal ischemic reperfusion injury^14^. Also, we found that the down-regulation of apo-A4 expression affected the recovery of kidney cells from ischemic reperfusion injury^14^. Thus, we hypothesized that kidney cell injury is influenced by increased apo-A4 expression level just after AKI. To test this hypothesis, we examined the role of apo-A4 and the apo-A4 associated pro-inflammatory pathway under conditions of AKI.

First, we observed endogenous apo-A4 expression levels in kidney cell lines and tissues. Apo-A4 has been reported to be expressed in proximal and distal tubular cells, capillaries, and blood vessels but not in the glomeruli of normal kidneys; however, we observed apo-A4 expression in kidney tubular cells as well as in the glomeruli of normal and injured kidney tissues^1, 4^. We anticipated apo-A4 accumulation in the kidney during ischemic reperfusion injury, and indeed, apo-A4 expression levels were elevated in CKD tissue compared with those in normal kidney tissue^4^. This result indicates that apo-A4 is up-regulated in response to kidney injury. The accumulation of apo-A4 has been reported in the blood plasma and urine of CKD patients during initial phases of the disease, suggesting that apo-A4 expression can predict the progression of kidney disease^2, 3, 5, 8^.

Also, we induced injury in human kidney cells using different AKI conditions. AKI is caused by decreased renal blood flow (ischemia of kidney tissue), exposure to harmful substances, inflammatory processes, or urinary tract obstructions, which impede the flow of urine^15, 16^. To mimic renal ischemia, we exposed human kidney cells to physical and chemical hypoxic conditions.

Additionally, we induced nephrotoxicity by treating kidney cells with cisplatin and induced apoptosis by treatment of kidney cells with the calcium ionophore (A23187), which increases intracellular Ca^2+^ levels^17, 18^. We did not observe any correlation between apo-A4 expression and renal injury model induced by cisplatin and A23187.

Finally, we investigated the effect of inflammatory injury on apo-A4 expression. Inflammatory processes comprise a critical response to pathogenic infection; however, the excessive production of inflammatory cytokines, such as TNF-α and IL-1β, may induce cell injury. TNF-α is a group of pro-inflammatory cytokines implicated in a wide variety of biological processes. This cytokine is produced by multiple cell types, including macrophages, lymphocytes, fibroblasts, and keratinocytes, as a response to inflammation, infection, and other environmental stresses^17, 19^. We found that the increase in apo-A4 expression level caused by TNF-α was related to pro-inflammatory AKI in human kidney cells. *In vivo*, we induced ischemic reperfusion injury in rat kidneys to investigate the effect of apo-A4 expression on specific conditions of inflammatory kidney injury. We invoked ischemic reperfusion injury using the clamping of renal hilar vessels (renal artery and renal vein) to elicit an inflammatory reaction in rat kidneys^7^. We provoked reperfusion injury and isolated protein lysates to measure apo-A4 expression levels by western blotting. Apo-A4 protein expression was elevated in ischemic reperfusion injury tissue compared with levels in control tissue (Fig. 3B). Also, we isolated serum samples from these rats and confirmed the increased apo-A4 levels by enzyme-linked immunosorbent assay (ELISA). Data presents 10samples were shown statistically significant (*P ≤ 0.05; Fig. 3C). *In vitro* and *in vivo*, these findings indicate that inflammatory AKI increases apo-A4 expression.

TNFR1 acts like cell death, altered target gene transcription, and cytokine production. TNFR2 interacts either in cooperation with or independently with TNFR1, acts as cell proliferation and cell survival. TNFR2 may have an antiapoptotic effect acting through an NF-kB pathway that results in exacerbated expression of several inflammatory mediators^20^. TNFR2 plays an important role during the inflammatory process^12, 19^. This receptor was up-regulated during intestinal inflammation, and concordantly, less intestinal inflammation was observed in TNFR2-deficient mice^11, 19, 21^. We demonstrated that apo-A4 expression was increased during inflammatory injury stimulated by TNF-α and that the expression of TNFR2 was increased in both kidney cell lines and experimented rat kidneys following AKI. Therefore, inflammatory injury stimulated by TNF-α induced TNFR2 activation.

TNF-α-mediated activation of the NF-κB complex initiates phosphorylation and proteasomal degradation of IκB within the cytoplasm, promoting nuclear translocation of the p65 subunit of NF-κB and enabling heterodimeric NF-κB to enter the nucleus. In the nucleus, NF-κB binds to enhancers of its target genes^12, 22, 23^. In our research, TNF-α inhibited IκB and activated NF-κB. Also, the expression of Apo-A4 decreased when IκB activity was high (Fig. 5A). TNF-α was found to be a major factor in the expression of apo-A4, because of the apo-A4 lower than the normal control when TNF-α antibody was administered.

We conduct an additional experiment that NF-κB regulates apo-A4 expression. It has reported that NF-κB inhibitors attenuate the inflammatory response in injured kidney animal model^24, 25^. Pyrrolidine dithio-carbamate ammonium (PDTC) is known to inhibit the NF-κB system and attenuated renal damage and inflammation^24, 25^. Apo-A4 was decreased when HK-2 cell treated by PDTC, compared to control group (Supplementary Fig. S4A,B). Apo-A4 was increased in the AKI model treated with TNF-α. The administration of TNF-α and PDTC in HK-2 cell attenuated apo-A4 expression (Supplementary Fig. S4A,B). Based on these results, we proved that NF-κB regulates apo-A4.

**Figure 6:**
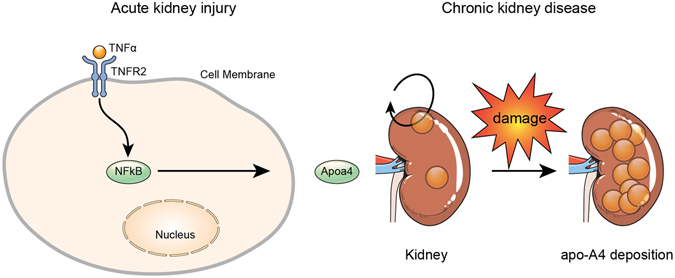
Proposed model of apo-A4 mediated signaling pathway in kidney injury. (**A**) TNF-α mediates an increase in apo-A4 expression during inflammatory AKI. TNF-α activates apo-A4 expression via the TNFR2-NFκB pathway. (**B**) Continuous kidney damage caused by ischemic reperfusion injury promotes apo-A4 deposition in kidney cells.

We showed that inflammatory damage induced TNFR2 activation via NF-κB in human kidney cell lines and tissues, and this finding is supported by the fact that the promoter region of TNFR2 contains binding sites for some transcription factors, including NF-κB. Taken together our results proposed that inflammatory AKI induced by TNF-α increases apo-A4 expression via the TNFR2-NFκB pathway (Fig. 6A).

Furthermore, we suggest that continuous kidney damage induced by inflammatory AKI disrupts apo-A4 metabolism, which promotes apo-A4 deposition in kidney cells, possibly leading to irreversible kidney injury or amyloidosis^4^. The mechanisms of apo-A4 function and metabolism in injured kidney tubular cells, however, remain unclear (Fig. 6B). Also, the factor that causes apo-A4 deposition during CKD requires identification^3, 4, 13^. Further investigation is needed to determine whether the protective role of apo-A4 is specific to NF-κB, and our results provide encouragement for further examinations of the therapeutic effects of apo-A4 in injured kidney cells.

In summary, we demonstrated that apo-A4 expression is increased by stimulation of injured kidney cells and tissues with TNF-α and that these effects occur via a TNFR2-NF-κB complex.

## Materials and Methods

### Human Tissues

Normal or ischemic human renal specimens were collected with the informed consent of patients according to the Institutional Review Board at Yonsei University (College of Medicine, Yonsei University), and the study was approved by the ethics committee. All human kidney biopsies were stored in liquid nitrogen.

### Animal Models

The study was approved by the Institutional Animal Care and Use Committee of Yonsei University Health System in accordance with the ‘Guide for the Care and Use of Laboratory Animals’ published by the National Institutes of Health and was conducted according to the principles of the Declaration of Helsinki(2015–0149). Eight-week-old male Sprague-Dawley rats were used for the renal ischemic reperfusion injury model. For the single kidney model, we performed right nephrectomy, and we initiated left kidney ischemia using renal hilar clamping for 30 min followed by reperfusion. Rats were sacrificed 48 h after ischemia-reperfusion of the kidney, and kidney tissues and blood samples were collected and stored in liquid nitrogen^7^.

### Cell Culture

HK-2 cells were cultured in keratinocyte serum-free medium (K-SFM, Gibco, Grand Island, NY, USA) including 0.05 mg/ml bovine pituitary extract, 5 ng/ml human recombinant epidermal growth factor, 10% fetal bovine serum, and 1% penicillin/streptomycin under an atmosphere of 5% CO2 and 95% O2 at 37 °C. Cells were subcultured every 7 days using 0.02% ethylenediaminetetraacetic acid and 0.05% trypsin. The medium was replaced with fresh medium every 2 days.

### Western blot

Total cellular protein extracts were prepared on ice using a PRO-PREP protein extract solution (Intron, Seoul, Korea). Cell lysates were loaded onto a sodium dodecyl sulfate-polyacrylamide gel and transferred to polyvinylidene fluoride membrane for 1 hour. Membranes were incubated at 4 °C overnight with HIF-1α, apo-A4, TNFR2, IκB, and α-tubulin primary antibodies (Abcam, Cambridge, MA, USA) diluted 1:1000 with 5% BSA Tris-buffered saline-Tween 20 (TBS-T). After incubation, the membranes were washed with TBS-T and then secondary antibodies (1:10000, horseradish peroxidase-conjugated anti-mouse IgG, anti-rabbit IgG) were added for incubation at room temperature for 1 hour. Labeled bands were detected by West Pico chemiluminescent kit (Thermo Scientific, Rockford, IL, USA).

### Immunofluorescence Assay

Cells were washed in phosphate-buffered saline (PBS) and fixed in 4% paraformaldehyde. Cells were subjected to permeation in 0.5% Triton X-100/ PBS for 5 min and then blocked in 5% BSA/PBS for 20 min. Cells were then incubated with mouse anti-apo-A4 antibody (Abcam) at 37 °C for 1 h and then incubated with Alexa Fluor 488 anti-mouse secondary antibody (1:200, Invitrogen) at 4 °C for 1 h. Images were acquired by confocal microscope (LSM Meta 700, Carl Zeiss, Oberkochen, Germany) and analyzed with LSM Image Browser software.

### IHC

Kidney tissue sections were incubated in formalin and treated overnight with 0.1 M citrate buffer antigen retrieval solution (pH 6.0; Dako, Carpinteria, CA, US) at 85 °C. Endogenous peroxidase was blocked with 3% H_2_O_2_ for 5 min. After blocking in 1% BSA in Tris-HCl buffer (BSA-T, pH 8.6) for 1 h, sections were incubated with the anti-human apo-A4 primary antibody (Abcam) diluted 1:400 in BSA-T for 1 h at room temperature. Then, specific immunoreaction was detected using a secondary horseradish peroxidase–conjugated goat anti-rabbit antibody (Dako). After washing in Tris-HCl buffer, 3-3-diaminbenzidine tetrahydrochloride (DAB + , Dako) was used for visualization. Sections were counterstained with hematoxylin, dehydrated, mounted in DePeX (Serva, Heidelberg, Germany), and examined with a microscope.

### ELISA

Rat plasma apo-A4 concentrations were determined using a double-antibody ELISA. The amounts of apo-A4 in the rat plasma were estimated using the apo-A4 rat ELISA kit (Mybiosource, Vancouver, Canada) according to the manufacturer’s instructions.

### Statistical analysis

Quantitative values were expressed as mean ± S.E.M. Statistical differences were compared using the Kruskal-Wallis test and Mann-Whitney U-test with SPSS software version 23.0 (IBM SPSS Statistics, IBM Corp., Armonk, N.Y., United States). *P* < 0.05 was considered statistically significant.

## Electronic supplementary material

Supplementary figures
