# Supplementary material for: TNF-α-induced Inflammation Stimulates Apolipoprotein-A4 via Activation of TNFR2 and NF-κB Signaling in Kidney Tubular Cells
Source: Sci Rep. 2017 Aug 18;7:8856. doi: 10.1038/s41598-017-08785-2 (PMC5562825; doi:10.1038/s41598-017-08785-2)
Supplement: Supplementary file 1 — Supplementary figures [file 41598_2017_8785_MOESM1_ESM.pdf]

SUPPLEMENTARY INFORMATION

TNF-α-induced Inflammation Stimulates Apolipoprotein-A4 via Activation of TNFR2 and NF-κB Signaling in Kidney Tubular Cells

Hyung Ho Lee[1,4], Young In Cho[1,2], Sook Young Kim[1], Young Eun Yoon[5], Kyung Sup Kim[2,3], Sung Joon Hong[1,2], Woong Kyu Han[1,2*]

[1] Department of Urology, Urological Science Institute, Yonsei University College of Medicine, Seoul, 120-752, Korea

[2] Brain Korea 21 PLUS Project for Medical Science, Yonsei University College of Medicine, Seoul, 120-752, Korea

[3] Department of Biochemistry and Molecular Biology, Institute of Genetic Science, Integrated Genomic Research Center for Metabolic Regulation, Yonsei University College of Medicine, Seoul, 120-752, Korea

[4] Department of Urology, National Health Insurance Service Ilsan Hospital, Gyeonggi-do, 10444, Korea

[5] Department of Urology, Hanyang Univesity College of Medicine, Seoul,04763, Korea

*Correspondence: Woong Kyu Han, MD, PhD

Department of Urology, Yonsei University College of Medicine

134 Shinchon-dong, Seodaemun-gu, Seoul, 120-752, Korea

Tel.: +82-2228-2325,   FAX: +82-312-2538,

E-mail: hanwk@yuhs.ac

Supplementary Figure

Figure S1

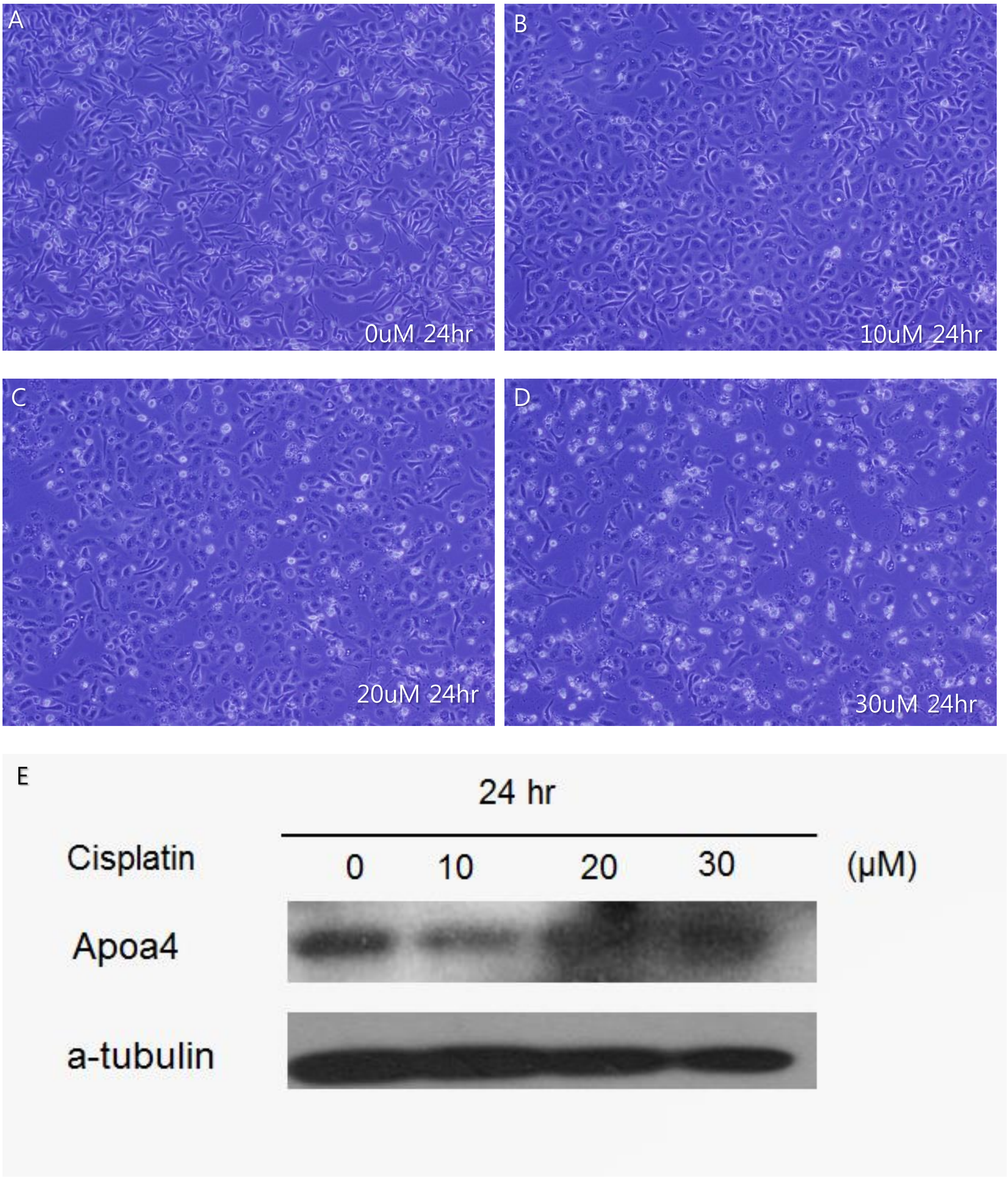

Figure S1. Cisplain induced acute kidney injury model.

We treated HK-2 cells with the nephrotoxic cancer drug cisplatin. HK2 were treated with various concentrations of cisplatin, as indicated, for 24 hours. Microscopic findings showed decreased cell viability with increasing cisplatin concentration(A, B, C, D). There was no significant difference in expression of apo-A4 in western blot (E).

Figure S2

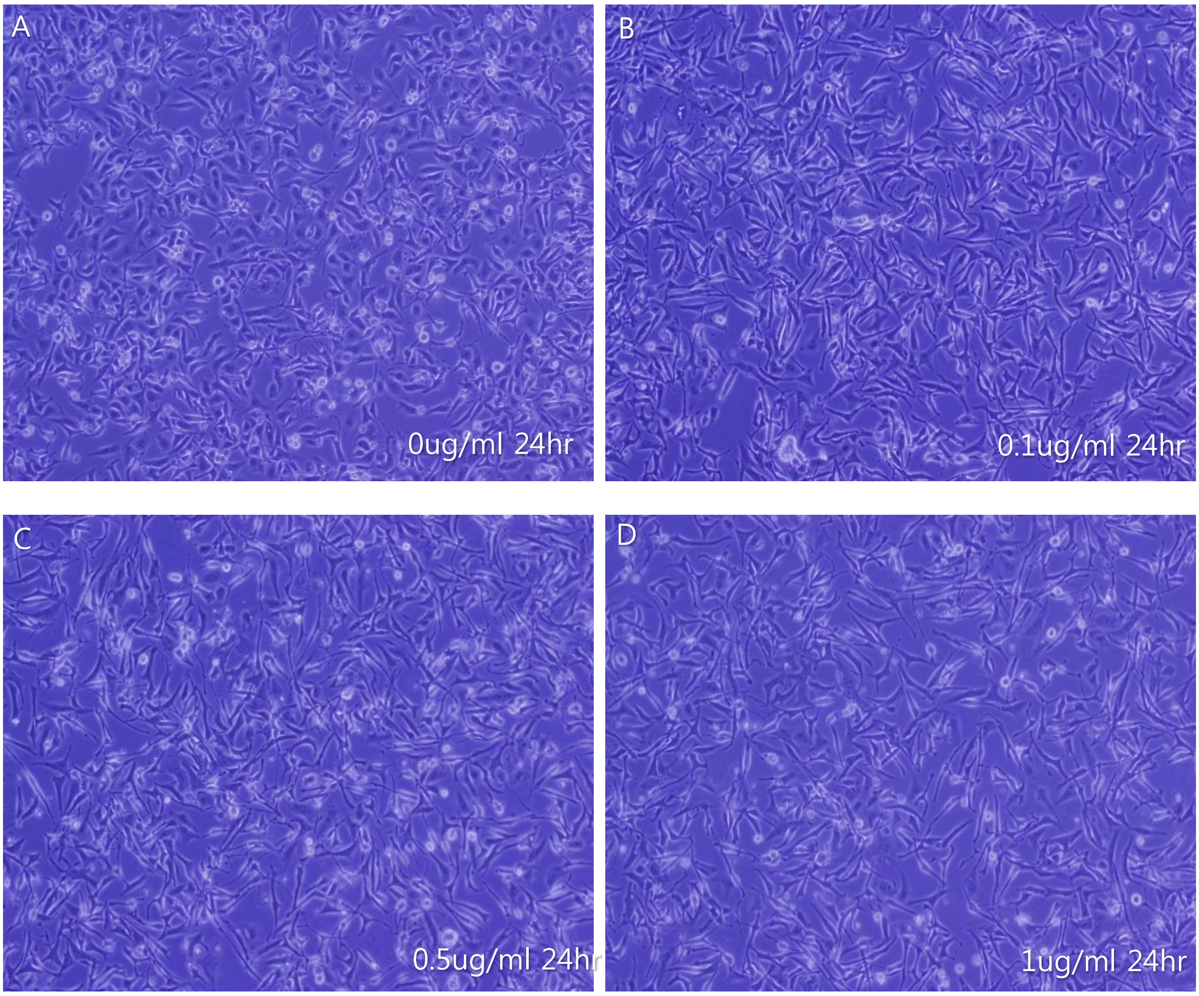
A
0ug/ml 24hr
B
0.1ug/ml 24hr
C
0.5ug/ml 24hr
D
1ug/ml 24hr

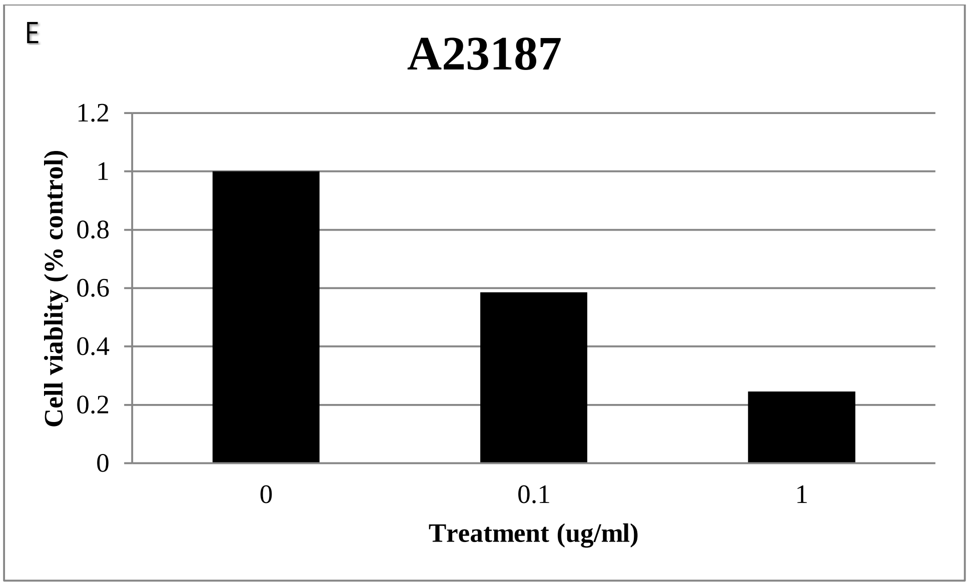
E
A23187
Cell viablity (% control)
1.2
1
0.8
0.6
0.4
0.2
0
0
0.1
1
Treatment (ug/ml)

Figure S2. Calcium ionophore A23187 induced acute kidney injury model.

We treated HK-2 cells with the Calcium ionophore A23187. HK2 were treated with various concentrations of A23187 for 24 hours Microscopic findings showed decreased cell viability with increasing cisplatin concentration(A, B, C, D). Cell viability was decreased in diagram(E).

Figure S3

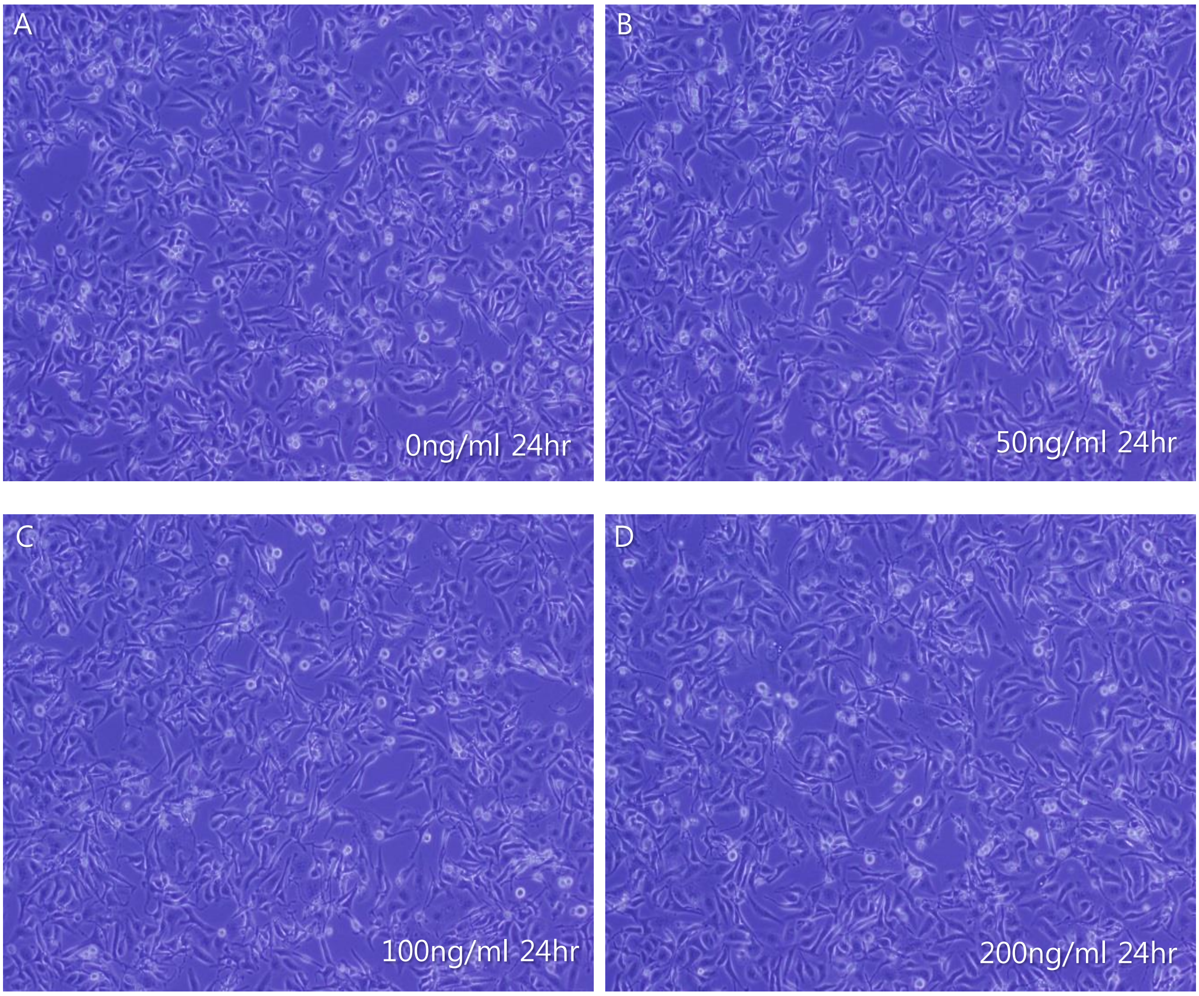
A
0ng/ml 24hr
B
50ng/ml 24hr
C
100ng/ml 24hr
D
200ng/ml 24hr

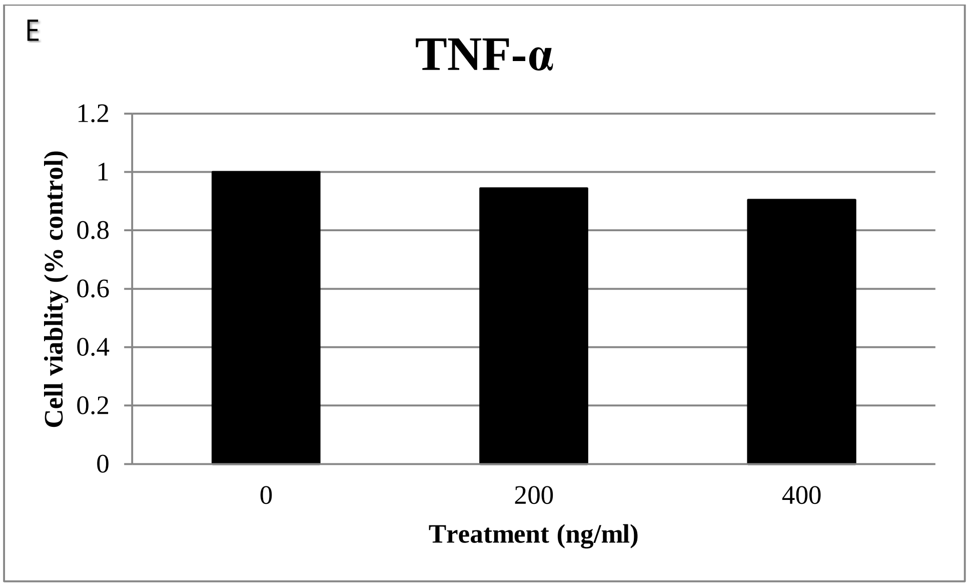
E
TNF-α
1.2
1
0.8
0.6
0.4
0.2
0
Cell viablity (% control)
0
200
400
Treatment (ng/ml)

Figure S3. TNF-α induced acute kidney injury model.

We treated HK-2 cells with the TNF-α. Microscopic findings showed cell viability reduction was less than cisplatin and A23187(A, B, C, D). Cell viability was less decreased in diagram(E).

Figure S4

A

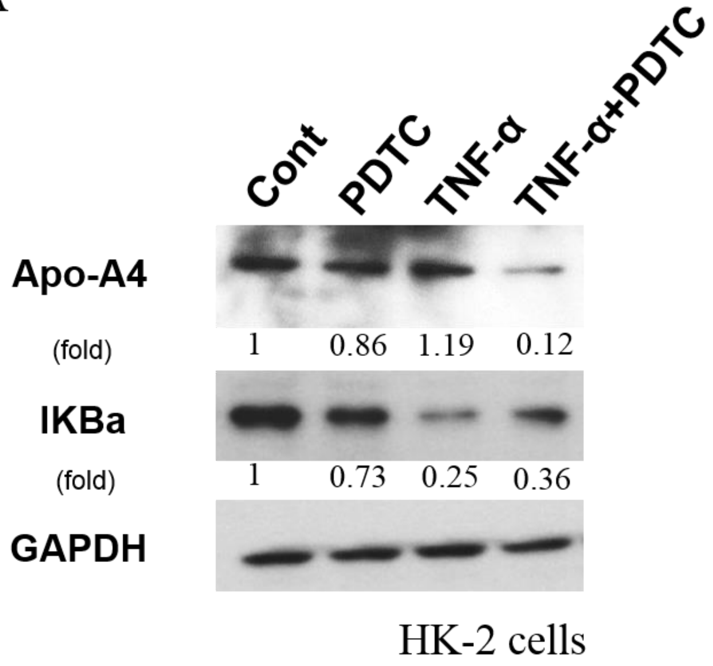
Cont
PDTC
TNF-α
TNF-α+PDTC
Apo-A4
(fold)
1
0.86
1.19
0.12
IKBa
(fold)
1
0.73
0.25
0.36
GAPDH
HK-2 cells

B

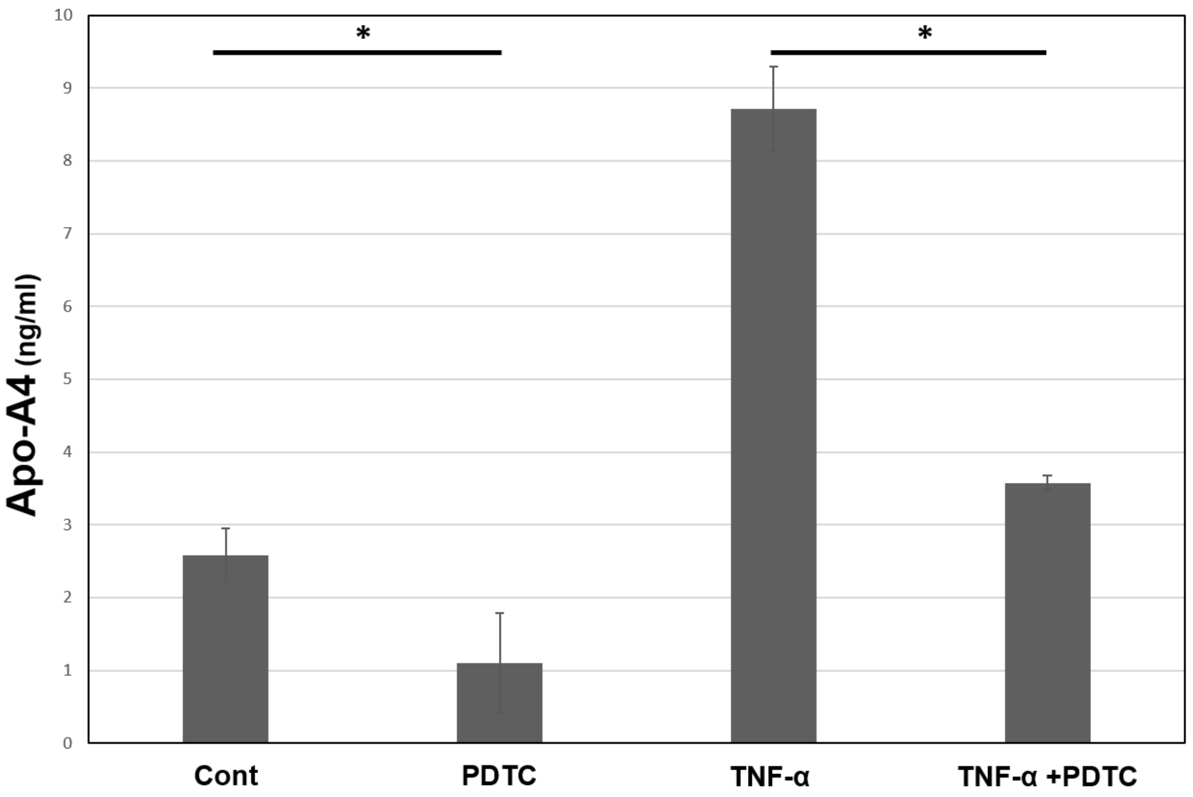
Apo-A4 (ng/ml)
10
9
8
7
6
5
4
3
2
1
0
*
*
Cont
PDTC
TNF-α
TNF-α +PDTC

Figure S4. NF-κB regulates apo-A4 expression

We treated HK-2 cells with the TNF-α and Pyrrolidine dithio-carbamate ammonium (PDTC). PDTC attenuated apo-A4 expression in western blot (Figure S4A). The expression of apo-A4 was reduced by PDTC(NF-κB inhibition) treated group (Figure S4B). In addition, apo-A4 was increased in the group treated with TNF-α, but apo-A4 expression was decreased when PDTC was administered, statistically (*p<0.05).
